# Maxillary Sinus in relation to Modern Oral and Maxillofacial Surgery

**DOI:** 10.1155/2012/391012

**Published:** 2012-12-06

**Authors:** Silvio Taschieri, Massimo Del Fabbro, Igor Tsesis, Stefano Corbella

**Affiliations:** ^1^Oral Health Research Centre, Department of Biomedical, Surgical and Dental Sciences, Università degli Studi di Milano, IRCCS Istituto Ortopedico Galeazzi, Milan, Italy; ^2^Department of Endodontology, Maurice and Gabriela Goldschleger School of Dental Medicine, Tel Aviv, Israel; ^3^Oral Implantology Research Centre, Department of Biomedical, Surgical and Dental Sciences, Università degli Studi di Milano, IRCCS Istituto Ortopedico Galeazzi, Milan, Italy

The maxillary sinus is a fundamental anatomical structure which is often involved in many oral and maxillofacial surgical procedures in the posterior maxilla, and whose integrity is important to preserve.

Infraction or invasion of maxillary sinus can occur during augmentation procedures and implant placement, especially when residual ridge height is reduced due to the process of bone loss after tooth extractions in the posterior maxilla. The invasion of maxillary sinus can hypothetically be considered a potential source of infection or irritation which can lead to inflammation of sinus membrane.

Because of these aspects, the placement of dental implants in the atrophic posterior maxilla is a challenging procedure in the presence of reduced maxillary bone height. 

Various techniques have been proposed in order to obtain the adequate bone dimension for the insertion of implants. However, due to the improvement of surgical techniques and the progress of research in the field of biomaterials, excellent outcomes have been reported in the last years for implant-supported rehabilitations even in cases with severe atrophy. 

Several types of complications may occur during and after the sinus elevation procedure with lateral approach. In fact, relatively frequent Schneiderian membrane perforations, nose bleeding, postoperative pain, and swelling could be considered as major drawbacks for this treatment alternative even though it was not described an important negative effect on implant success rates.

In this special issue, several aspects about implant placement in relation to interventions to augment posterior maxilla bone volume were discussed, and the reader can found a summary of the contents of the articles included. 

The results of a clinical consensus of experts published in this issue (periodontists, implantologist, maxillofacial surgeons, ENT, and microbiology specialists) on several clinical questions and to give clinical recommendations on how to prevent, diagnose, and treat postoperative infections, can be useful for the clinician to make the right treatment choice and give guidelines for handling the intra-operative and post-operative complications. Moreover, the presented guidelines showed that a multidisciplinary approach can help in limiting the occurrence of complications and improving patients' quality of life.

S. A. Gehrke et al. showed a repair technique of a perforated sinus membrane with a subepithelial palatal conjunctive flap. Authors concluded that the use of conjunctive technique with collected palate flap for sealing the perforation of the membrane of the sinus may have predictable result. It could be hypothesized that repairing the sinus injury entrapping the bone graft in a safety and closed sinus cavity, and achieving a contact between this package and the vascularized sinus walls could be enough to favor angiogenesis and contextually the developing of newly formed vital bone. 

M. Beretta et al. highlight the correct steps for doing sinus lift surgery in presence of anatomic variations such as sinus septa. Radiographic identification of these structures is important in order to perform the right design of the lateral window during sinus lift and to avoid complications related to the sinus lift surgery. The correct steps in performing the surgical procedures can be an important aid in the sinus lifting procedure with lateral approach in presence of sinus septa.

N. Cavalli et al. in this issue presented an alternative technique in case of severe atrophic posterior maxilla. Patients received a maxillary full-arch fixed bridge supported by two axial implants and two distal tilted implants, without a sinus lift management. The overall follow-up range was 12 to 73 months (mean 38.8 months). The high cumulative implant survival rate indicates that this technique could be considered a viable treatment option. Authors underlined the relevance of an effective recall program in order to early intercept and correct prosthetic and biologic complications in order to avoid implant and prosthetic failures. It should be underlined that this procedure can be useful to avoid the management of maxillary sinus in cases of the presence of sinus pathology.

While autogenous bone has long been considered the gold standard grafting material because of its osteoinductive and osteoconductive properties, alternative materials have, in general, no osteoinductive potential but are considered to provide a scaffold for optimal bone growth. The efficacy of the graft material in promoting graft maturation and providing optimal long-term support to endosseous implants is one of the most critical factors for the success of the sinus augmentation procedure.

A. Troedhan et al. investigated the key role of the sinus-membrane in bone reformation in vivo. The results of this study proved the key role of the sinus-membrane as the main carrier of bone-reformation after sinuslift-procedures as multiple experimental studies suggested. Thus the importance of minimal invasive and rupture free sinuslift procedures is underlined and does not depend on the type of grafting material used.

F. Riachi et al. investigated the influence of material properties on rate of resorption of two bone graft materials after sinus lift using radiographic assessment showing that the chemical and physical properties of bone graft material significantly influence resorption rate of bone graft materials used for sinus augmentation.

The aim of the study presented by S. Taschieri et al. was to systematically review the existing literature on transalveolar maxillary sinus augmentation without grafting materials and to propose and describe an osteotome-mediated approach in postextraction sites in combination with platelet derivative. 

While transcrestal approach is considered more conservative than a lateral approach, the main drawback is that the main part of the sinus lifting procedure must be performed blindly because of the impossibility to visualize the sinus floor. Considering this limitation, the systematic review showed that high implant survival rate (more than 96% after 5 years) can be achieved even without grafting the site, with a low rate of complications. Available alveolar bone height before surgery was not correlated to survival rate. The osteotome-mediated sinus lifting technique was performed with the use of platelet derivative (PRGF). The presented technique might represent a viable alternative for the treatment of edentulous atrophic posterior maxilla, more than 5 mm of residual bone height, of the alveolar bone though it needs to be validated by studies with a large sample size. 

In general, sinus lifting through a lateral approach is a viable technique when less than 4-5 mm of residual bone height is present. When more than 5 mm of residual bone height is available a transalveolar approach, with or without adding bone substitute, or the insertion of short implant could be indicated as alternatives techniques in order to reduce the morbidity and the invasiveness of the treatment protocol. The choice of the most suitable technique among the three ones above mentioned depends on the sinus physiology, the patient's desire to attempt a long and challenging rehabilitation with respect to a shorter one and the patient's financial status. 

The analysis of the data of the literature suggested that short implants, osteotome-mediated sinus floor elevation (with or without bone substitute) and lateral approach sinus floor elevation had similar clinical outcomes and appeared to be comparable. A wider literature was available for lateral approach sinus floor elevation while short implants should be supported by more well-designed studies with a detailed description of implant demographics.

